# Circulating 27-hydroxycholesterol and breast cancer tissue expression of CYP27A1, CYP7B1, LXR-β, and ERβ: results from the EPIC-Heidelberg cohort

**DOI:** 10.1186/s13058-020-1253-6

**Published:** 2020-02-19

**Authors:** Charlotte Le Cornet, Britta Walter, Disorn Sookthai, Theron S. Johnson, Tilman Kühn, Ester Herpel, Rudolf Kaaks, Renée T. Fortner

**Affiliations:** 1 Division of Cancer Epidemiology, German Cancer Research Center (DKFZ), Im Neuenheimer Feld 280, 69120 Heidelberg, Germany; 2 Institute of Pathology, University Hospital Heidelberg, Im Neuenheimer Feld 224, 69120 Heidelberg, Germany; 3 Tissue Bank of the National Center for Tumor Diseases (NCT), Im Neuenheimer Feld 460, 69120 Heidelberg, Germany

**Keywords:** 27-hydroxycholeterol, Tissue microarray, Immunohistochemistry, Cholesterol metabolism, Breast cancer

## Abstract

**Background:**

Experimental and epidemiological studies demonstrate a role for 27-hydroxycholesterol (27HC) in breast cancer development, though results are conflicting. Cholesterol 27-hydroxylase (CYP27A1) and oxysterol 7-alpha-hydroxylase (CYP7B1) regulate 27HC concentrations, while differential expression of the liver X receptor (LXR) and estrogen receptor beta (ERβ) may impact the association between 27HC and breast cancer risk.

**Methods:**

We evaluated correlates of tumor tissue expression of CYP27A1, CYP7B1, LXR-β, and ERβ and the association between circulating prediagnostic 27HC concentrations and breast cancer risk by marker expression in a nested case-control study within the European Prospective Investigation into Cancer and Nutrition (EPIC)-Heidelberg cohort including 287 breast cancer cases with tumor tissue available. Tumor protein expression was evaluated using immunohistochemistry, and serum 27HC concentrations quantified using liquid chromatography–mass spectrometry. Conditional logistic regression models were used to estimate odds ratios (ORs) and 95% confidence intervals (CIs).

**Results:**

A higher proportion of CYP7B1-positive cases were progesterone receptor (PR)-positive, relative to CYP7B1-negative cases, whereas a higher proportion of ERβ-positive cases were Bcl-2 low, relative to ERβ-negative cases. No differences in tumor tissue marker positivity were observed by reproductive and lifestyle factors. We observed limited evidence of heterogeneity in associations between circulating 27HC and breast cancer risk by tumor tissue expression of CYP27A1, CYP7B1, LXR-β, and ERβ, with the exception of statistically significant heterogeneity by LXR-β status in the subgroup of women perimenopausal at blood collection (*p* = 0.02).

**Conclusion:**

This exploratory study suggests limited associations between tumor marker status and epidemiologic or breast cancer characteristics. Furthermore, the association between circulating 27HC and breast cancer risk may not vary by tumor expression of CYP27A1, CYP7B1, LXR-β, or ERβ.

**Electronic supplementary material:**

The online version of this article (10.1186/s13058-020-1253-6) contains supplementary material, which is available to authorized users.

## Introduction

27-Hydroxycholesterol (27HC) is an abundant oxysterol in blood and plays an intermediate role in cholesterol catabolism to bile acids. Two key enzymes produced from cytochrome P450 genes are involved in 27HC regulation: cholesterol 27-hydroxylase (CYP27A1) and oxysterol 7-alpha-hydroxylase (CYP7B1). CYP27A1 is responsible for conversion of cholesterol into 27HC whereas CYP7B1 catabolizes 27HC toward bile acid synthesis [1]. Experimental studies identified 27HC as an endogenous selective estrogen receptor modulator (SERM) [2]. 27HC binds to both the estrogen receptor alpha (ERα) and estrogen receptor beta (ERβ) [2, 3], though with greater affinity for ERβ [3]. Although the precise roles of ERβ in breast cancer remain to be delineated [4], ERβ was demonstrated to be expressed in a majority of breast cancers, including those lacking ERα expression. Independent from estrogen receptor (ER)-mediated actions, 27HC is a liver X receptor (LXR) ligand [5] and has been implicated in breast cancer metastasis via the LXR in experimental animal models [6].

The role of 27HC in the etiology and progression of breast cancer has been investigated in experimental animal models [6, 7], with limited data in epidemiologic studies and patient populations to date [8–10]. In experimental cell-line models, 27HC induced cell proliferation through ER activation, though administration of 27HC reduced estradiol-induced proliferation [2]. Higher 27HC was associated with disease progression in experimental animal models [6, 7]. One prospective trial in breast cancer patients reported a significant increase of 27HC in response to aromatase inhibitor but not to tamoxifen treatment [9]. Our group previously published on prediagnosis circulating 27HC and breast cancer risk, reporting an inverse association between circulating 27HC and breast cancer risk in postmenopausal women and no significant heterogeneity by tumor ERα status (clinically measured) [8]. Higher tumor *CYP27A1* mRNA expression has been associated with better prognosis in women 50 or younger and with ERα-positive breast cancer [10], though other studies have not observed an association in a broader population [6, 7]. *CYP7B1* expression has been shown to be lower in ERα-positive breast tumors, relative to normal breast tissue [7], and associated with better prognosis [6, 7]. Correlates of tumor protein expression of CYP27A1 and CYP7B1 with cancer characteristics, reproductive and lifestyle factors are not well established in cancer [6, 10, 11], including breast cancer [6, 10].

In the context of recent evidence linking circulating 27HC to breast cancer risk, prior data on 27HC and breast cancer progression, and relationships between circulating 27HC and CYP27A1, CYP7B1, LXR-β, and ERβ, the aims of the present study were to investigate the associations between (i) protein expression of these markers in the breast tumor tissue and breast cancer case characteristics, (ii) protein expression of these markers in the breast tumor tissue and epidemiological factors and circulating sex steroids and lipids, and (iii) prediagnostic 27HC concentrations in blood and breast cancer risk by these markers. This was an exploratory study in which we hypothesized potential differential protein expression by case characteristics (e.g., CYP27A1 associated with favorable prognostic characteristics such as tumor grade), and heterogeneity in associations between 27HC and breast cancer risk by tumor protein expression (e.g., stronger association between 27HC and breast cancer among cases with CYP27A1-positive tumors). This study was conducted within the Heidelberg, Germany cohort of the European Investigation into Cancer and Nutrition (EPIC).

## Materials and methods

### Study population: the EPIC-Heidelberg cohort

The EPIC-Heidelberg cohort includes 25,540 study participants (13,611 women) recruited between 1994 and 1998 with between ages 35 and 65 years. Blood samples, anthropometric, reproductive, and epidemiologic factors including diet and lifestyle behavior were collected at baseline and described previously [12, 13]. Blood samples are stored under liquid nitrogen (− 196 °C) at the German Cancer Research Center (DKFZ, Heidelberg, Germany).

### Case identification

All incident cases of invasive breast cancer were identified and verified by study physicians during prospective follow-up (through January 2013), by active follow-up with study participants or derived from hospital records, or cancer and mortality registries. Each participant case was free of any previous invasive cancer (with the exception of non-melanoma skin cancer) or in situ breast cancer history prior to invasive breast cancer diagnosis, and had a blood sample available collected at baseline. Overall, 530 cases met these criteria, including 287 (54.1%) for which tumor blocks were available and who were included on a tissue microarray (TMA).

### Nested case-control study

Up to two matched controls per case were selected from female participants in EPIC-Heidelberg with a blood sample collected at baseline and who remained cancer free (with the exception of non-melanoma skin cancer) to the time of diagnosis of their matched case. Controls were matched to cases based on age (± 3 months), menopausal status, exogenous hormones use, fasting status (< 3; 3–6; > 6 h), and time of the day (±1 h) at blood donation. Premenopausal women were also matched for phase of menstrual cycle (early follicular, late follicular, peri-ovulatory, early luteal, mid luteal, late luteal). The 287 cases included in this study were matched to 563 controls (11 cases were matched to one control only); however, all 1036 controls from the parent study were included in the current study, with statistical analyses adjusting for the matching factors. All participants provided informed consent and the study was approved by the ethics commission of the Heidelberg University Medical Faculty (reference numbers: 13/94, S-551/2014).

### Data collection

Tumor characteristics such as clinically measured estrogen (ER) and progesterone (PR) receptor status, breast cancer stage, grade, and morphology were extracted from clinical records by trained study physicians. Baseline information on reproductive and lifestyle factors was obtained from standardized self-administered questionnaires and computer-assisted interview at recruitment. Ever full-term pregnancy (FTP; no, yes), number of FTPs (0, 1, 2, ≥ 3), use of oral contraceptives (OC) or postmenopausal hormone therapy (HT), menopausal status (premenopausal, perimenopausal, postmenopausal (includes surgical menopause, *n* = 6)), ever breast-feeding (no, yes), smoking status (never, former, current), and lifetime alcohol use at recruitment (never users, former users, and lifetime users with an average intake ≥ 6, > 6–12, > 12–24, > 24–60 g/d) were available from the baseline questionnaire. Lifetime alcohol use was assessed based on the alcoholic beverage consumption during the 12 months before recruitment and the consumption reported for ages 20, 30, 40, and 50 years. Anthropometric factors were measured and recorded at baseline by trained personnel [13]. Body mass index (BMI) was categorized according to the World Health Organization definition (kg/m^2^: thin < 18.5, normal 18.5–25; overweight 25–30; obese ≥ 30) based on height and weight. The two lowest categories were combined, as only one participant had BMI < 18.5. The Cambridge Index of physical activity was used; this index combines occupational and recreational physical activity and is categorized into four groups (active, moderately active, moderately inactive, and inactive). Additional details on baseline data collection have been described previously [12, 13].

### Tissue collection and immunohistochemistry

Collection of tumor tissue has been previously described [14]. For the current study, formalin-fixed paraffin-embedded (FFPE) breast tumor tissue material was available for 287 cases. Overall, cases included on the tissue microarrays (TMAs) were younger at diagnosis (59.1 versus 61.9 years, *p* < 0.01) and were more likely to have been diagnosed with higher-grade tumor (*p* = 0.008) and ductal morphology (*p* < 0.01), compared to cases not included on the TMAs (Additional file 1: Table S1). There were no significant differences regarding reproductive, anthropometric, and lifestyle factors, or for blood biomarkers, including circulating 27HC concentration.

TMA slides were prepared by taking two cores of 1 mm from a representative section of tumor for each case. Immunohistochemistry (IHC) staining was performed on TMA slides at the Tissue Bank of the National Center for Tumor Diseases (NCT), Heidelberg, Germany. A single pathology resident (B.W.) evaluated protein expression of CYP27A1, CYP7B1, LXR-β, and ERβ. Each case had two cores on the TMA; the highest score of the two cores was used to determine staining positivity. Immunostaining was scored either for cytoplasmic (CYP27A1, CYP7B1) or nuclear (LXR-β, ERβ) expression. Staining intensity in tumor cells was scored as 0 (absent), 0.5 (borderline), 1 (weak), 2 (moderate), or 3 (strong). Tumor cells were considered positive for CYP7B1 [15] and LXR-β when more than 10% of the cells showed moderate or strong staining. ERβ was considered positive when moderate or strong staining was observed for more than 50% of tumor cells [16]. For CYP27A1, any moderate to strong tumor cell staining was considered positive [6]. Table 1 shows antibodies and dilutions used and the definitions applied to determine tumor marker positivity. Where a conclusive result for a marker was not available, that marker was categorized as missing (CYP27A1, 4.8%; CYP7B1, 15%). For one slide, CYP7B1 staining failed, resulting in the exclusion of 43 cases for that marker. Additionally, the expression of Ki67 proliferation activity (low, high), Bcl-2 (negative, positive), and p53 (negative, positive) were also assessed in these cases, as previously described [14]. Pathology personnel were blinded to biomarker status.

**Table 1:** Immunohistochemistry (IHC) of breast tumor marker expression distribution: EPIC-Heidelberg nested case-control study

Marker	Antibody	Dilution	Defined as:		*N* = 287	%
CYP27A1	Abcam		Positive = moderate or strong (2 or 3) intensity in cytoplasm; otherwise negative	–	201	73.6
	(EPR7529)	1:50		+	72	26.4
CYP7B1	Abcam (ab19043)	1:500	Positive = moderate or strong (2 or 3) intensity in cytoplasm of ≥ 10% of cells; otherwise negative	–	157	64.6
				+	86	35.4
LXR-β	Abcam (ab24361)	1:25	Positive = moderate or strong (2 or 3) intensity in nucleus of ≥ 10% of cells; otherwise negative	–	123	42.9
				+	164	57.1
ERβ	Serotec (PPG5/10)	1:500	Positive = moderate or strong intensity (2 or 3) nuclear staining in > 50% tumor cells; otherwise negative	–	84	29.3
				+	203	70.7

Missing due to inconclusive staining: 44 for CYP7B1, 14 for CYP27A1

287 matched pairs of cases and controls have been used for the analysis (243 breast cancer cases have no tumor blocks available)

### Laboratory assays

Serum 27HC concentrations were quantified using liquid chromatography–mass spectrometry (LC-MS) at Biocrates Life Sciences (Innsbruck, Austria); lipid biomarkers (cholesterol, triglycerides, high-density lipoproteins, and low-density lipoproteins) were quantified by Synlab MVZ Heidelberg GmbH (Eppelheim, Germany); and sex steroids (testosterone, progesterone, estradiol, estrone, and dehydroepiandrosterone sulfate (DHEAS)) were measured in the Division of Cancer Epidemiology labs at the DKFZ. Case and control sets were analyzed together within the analytic batch, with case and control position randomized within the set. Laboratory personnel were blinded to case or control status, and two blinded pooled quality control (QC) samples were included to monitor the assay precision. Details on the measurement of the blood-based biomarkers have been described previously [8].

### Statistical analysis

Blood biomarkers (27HC, lipids, and sex steroids) were log2-transformed to better fit a normal distribution. Fisher’s exact test (categorical variables) and Welch’s *t*-test (continuous variables) were used to compare tumor characteristics, reproductive, anthropometric, and lifestyle factors, as well as circulating lipid and sex steroid levels by CYP27A1, CYP7B1, LXR-β, and ERβ tumor marker status (positive, negative). Analyses of sex steroid hormones were stratified by menopausal status and by HT use among woman postmenopausal at blood collection. We used menstrual cycle phase-specific residuals for estrone, estradiol, and progesterone to account for within-person variability in these hormones across the menstrual cycle among postmenopaual women. Odds ratios (ORs) and 95% confidence intervals (CIs) were estimated using unconditional logistic regression models, adjusted for the study matching factors, stratified by tumor marker status. Additionally, ORs were adjusted for age at first menstrual period, BMI, age at first full-term pregnancy, and number of FTP since adjustment for additional variables had minimal impact on the ORs in the parent study [8]. A total of 8 women had missing values for both fasting status and time at blood draw (1 case and 7 controls). These women were excluded from the risk analyses; thus, the sample used for these analyses included 286 cases and 1029 controls. Further, analyses were stratified by menopausal status given significant heterogeneity in associations between circulating 27HC and breast cancer risk by menopausal status was previously observed [8]. Heterogeneity by tumor marker status (positive vs. negative) was assessed using polytomous logistic regression models comparing models assuming the same association versus different associations in breast cancer in subgroups [17]; models were compared using a likelihood ratio test. We evaluated heterogeneity by menopausal status at blood collection by including an interaction term and evaluating the Wald test. Statistical analyses were conducted using SAS software, version 9.3 (SAS Institute, Cary, NC, USA). *P* values are two-sided, and *p* < 0.05 was considered statistically significant.

## Results

All descriptive analyses were made on the 287 breast cancer cases for whom tumor tissue was available. The average age at recruitment for cases was 51.4 years (standard deviation ± 7.9) and 51.2 years (± 7.9) for controls, and blood samples were collected in average 8 years [range 0.06–15.95] before breast cancer diagnosis.

### Associations between CYP27A1, CYP7B1, ERβ, and LXR-β expression with tumor and epidemiologic characteristics

A total of 26.4% of the cases were classified as positive for CYP27A1, 35.4% were classified as positive for CYP7B1, 57.1% were classified as positive for LXR-β, and 70.7% were classified as positive for ERβ. A higher proportion of CYP27A1-positive tumors and LXR-β-positive tumors were also ERβ-positive (*p* < 0.01) (Additional file 2: Table S2), relative to those negative for the respective markers. No other statistically significant associations between the four evaluated markers were observed (*p* ≥ 0.16).

No significant associations was observed between CYP27A1 or LXR-β expression and case characteristics (Table 2), though the proportion of cases with high expression of p53 was suggestively higher in CYP27A1-positive vs. CYP27A1-negative tumors (88.4% vs. 77.6%, *p* = 0.05). Differences in tumor characteristics by CYP7B1 and ERβ expression were minimal. A higher proportion of PR-positive (*p* = 0.04) tumors was observed in CYP7B1-positive tumors, in comparison with CYP7B1-negative tumors (PR-positive: 83.7% vs. 72.0%, respectively). A higher proportion of Bcl2_low_ expression (66.7% vs. 49.3%, *p* = 0.01) was observed in ERβ-positive compared to ERβ-negative tumors. No associations were observed between the investigated markers and other breast cancer characteristics (*p* ≥ 0.06), including age at diagnosis and cancer stage, grade, or morphology, or with any of the investigated epidemiologic factors (*p* ≥ 0.07, e.g., BMI, number of term pregnancies, menopausal status (Additional file 3: Table S3)). No associations between the tumor markers and serum 27HC were observed (Additional file 4: Table S4). Circulating high-density lipoproteins differed significantly by tumor CYP27A1 expression (*p* = 0.04; geometric means, CYP27A1-positive 64.3 mg/dl, CYP27A1-negative 68.8 mg/dl) and triglycerides differed significantly by tumor CYP7B1 expression (*p* = 0.02; geometric means, CYP7B1-positive 106.4 mg/dl, CYP7B1-negative 123.1 mg/dl). No consistent patterns were observed for sex steroid hormones, though higher circulating premenopausal DHEAS was observed among women with CYP27A1-positive compared to CYP27A1-negative tumors (*p* = 0.04), and higher circulating premenopausal estradiol (*p* = 0.02) and lower perimenopausal testosterone (*p* = 0.03) were observed among women with CYP7B1-positive compared to CYP7B1-negative tumors.

**Table 2:** Breast cancer characteristics by tumor marker status: EPIC-Heidelberg nested case-control study

	All cases	CYP27A1			CYP7B1			LXR-β			ERβ		
		Negative	Positive	*p*^a^	Negative	Positive	*p*^a^	Negative	Positive	*p*^a^	Negative	Positive	*p*^a^
*N*	287	201	72		157	86		123	164		84	203	
Age at diagnosis		58.84 ± 7.69	59.35 ± 8.81	0.71	59.22 ± 8.23	60.51 ± 7.66	0.28	58.76 ± 8.08	59.39 ± 8.01	0.57	60.12 ± 7.59	58.68 ± 8.20	0.22
Receptor subtype													
ER+	236	166 (83.0)	58 (80.6)	0.72	129 (82.2)	74 (86.0)	0.47	102 (82.9)	134 (82.2)	1.00	68 (81.0)	168 (83.2)	0.73
ER−	50	34 (17.0)	14 (19.4)		28 (17.8)	12 (14.0)		21 (17.1)	29 (17.8)		16 (19.0)	34 (16.8)	
ER+/PR+	206	142 (82.6)	52 (81.3)	0.85	111 (81.0)	69 (88.5)	0.18	87 (82.9)	119 (82.6)	1.00	59 (80.8)	147 (83.5)	0.59
ER−/PR−	43	30 (17.4)	12 (18.8)		26 (19.0)	9 (11.5)		18 (17.1)	25 (17.4)		14 (19.2)	29 (16.5)	
PR+	213	146 (73.0)	54 (75.0)	0.88	113 (72.0)	72 (83.7)	0.04	90 (73.2)	123 (75.5)	0.68	61 (72.6)	152 (75.2)	0.66
PR−	73	54 (27.0)	18 (25.0)		44 (28.0)	14 (16.3)		33 (26.8)	40 (24.5)		23 (27.4)	50 (24.8)	
HER2+	46	36 (18.0)	9 (12.5)	0.36	26 (16.6)	14 (16.3)	1.00	19 (15.4)	27 (16.6)	0.87	14 (16.7)	32 (15.8)	0.86
HER2−	240	164 (82.0)	63 (87.5)		131 (83.4)	72 (83.7)		104 (84.6)	136 (83.4)		70 (83.3)	170 (84.2)	
ER−/PR−/HER2−	31	19 (9.5)	11 (15.3)	0.19	17 (10.8)	6 (7.0)	0.21	12 (9.8)	19 (11.7)	0.70	9 (10.7)	22 (10.9)	1.00
ER+ or PR+ or HER2+	255	181 (90.5)	61 (84.7)		140 (89.2)	80 (93.0)		111 (90.2)	144 (88.3)		75 (89.3)	180 (89.1)	
Ki67													
Low	46	34 (17.7)	12 (18.5)	0.85	23 (16.3)	8 (9.6)	0.23	15 (14.0)	31 (19.6)	0.25	11 (15.1)	35 (18.2)	0.59
High	219	158 (82.3)	53 (81.5)		118 (83.7)	75 (90.4)		92 (86.0)	127 (80.4)		62 (84.9)	157 (81.8)	
bcl2													
Low	165	121 (62.7)	40 (59.7)	0.66	79 (56.4)	54 (64.3)	0.26	71 (64.5)	94 (60.3)	0.52	35 (49.3)	130 (66.7)	0.01
High	101	72 (37.3)	27 (40.3)		61 (43.6)	30 (35.7)		39 (35.5)	62 (39.7)		36 (50.7)	65 (33.3)	
p53													
Low	52	43 (22.4)	8 (11.6)	0.05	31 (22.0)	12 (14.3)	0.17	19 (17.6)	33 (20.9)	0.53	13 (18.3)	39 (20.0)	0.86
High	214	149 (77.6)	61 (88.4)		110 (78.0)	72 (85.7)		89 (82.4)	125 (79.1)		58 (81.7)	156 (80.0)	
Breast cancer stage													
Local	175	119 (61.0)	44 (61.1)	0.46	94 (60.6)	56 (68.3)	0.53	82 (67.2)	93 (58.5)	0.12	43 (53.1)	132 (66.0)	0.07
Regional	100	73 (37.4)	25 (34.7)		57 (36.8)	25 (30.5)		36 (29.5)	64 (40.3)		35 (43.2)	65 (32.5)	
Metastasis	6	3 (1.5)	3 (4.2)		4 (2.6)	1 (1.2)		4 (3.3)	2 (1.3)		3 (3.7)	3 (1.5)	
Breast cancer grade													
Grade I	38	30 (15.0)	7 (9.9)	0.41	21 (13.4)	15 (17.6)	0.64	18 (14.8)	20 (12.3)	0.06	7 (8.3)	31 (15.4)	0.28
Grade II	163	114 (57.0)	39 (54.9)		87 (55.4)	43 (50.6)		77 (63.1)	86 (52.8)		50 (59.5)	113 (56.2)	
Grade III	84	56 (28.0)	25 (35.2)		49 (31.2)	27 (31.8)		27 (22.1)	57 (35.0)		27 (32.1)	57 (28.4)	
Breast cancer morphology													
Ductal	202	142 (70.6)	54 (75)	0.19	109 (69.4)	59 (68.6)	0.68	83 (67.5)	119 (72.6)	0.21	61 (72.6)	141 (69.5)	0.48
Lobular	56	36 (17.9)	15 (20.8)		29 (18.5)	19 (22.1)		23 (18.7)	33 (20.1)		13 (15.5)	43 (21.2)	
Other	29	23 (11.4)	3 (4.2)		19 (12.1)	8 (9.3)		17 (13.8)	12 (7.3)		10 (11.9)	19 (9.4)	

^a^Fisher’s exact tests for categorical variable [*n* (%)] or Welch’s *t*-test for continuous variable [mean ± std]; Missing: ER = 1, PR = 1, HER2 = 1, TNBC = 1, breast cancer stage = 6, breast cancer grade = 2, Ki67 = 22, bcl2 = 21, p53 = 21, CYP7B1 = 44, CYP27A1 = 14

### Circulating 27HC and breast cancer risk by tumor markers

We observed no statistically significant heterogeneity in associations between circulating 27HC and breast cancer risk by tumor expression of CYP27A1, CYP7B1, LXR-β, or ERβ (Table 3). Heterogeneity in associations between circulating 27HC and breast cancer risk by menopausal status at blood collection was only observed in tumor not expressing LXR-β (*p*_het_ = 0.02). This was driven by a significant positive association observed among perimenopausal women; however, sample size was limited in this subgroup (*n* = 54 total perimenopausal cases). In postmenopausal women, higher 27HC concentrations were associated with lower risk of ERβ-negative breast cancer (OR_log2_:0.31; 95%CI 0.10, 0.92; *p*_het_ by menopausal status in ERβ-negative = 0.06), whereas no association was observed among ERβ-positive cases (OR_log2_: 0.72 (95% CI 0.36, 1.47); *p*_het_ by ERβ status in postmenopausal = 0.12) (*n* = 146 postmenopausal cases with ERβ data).

**Table 3:** Prediagnosis circulating 27HC and breast cancer risk by breast tumor expression of select IHC markers: EPIC-Heidelberg nested case-control study

			Menopausal status at blood collection			
		All women	Premenopausal	Perimenopausal	Postmenopausal	*p*_het_^b^
All women	Cases/controls	286/1029	86/315	54/195	146/519	
	OR (95% CI)^a^	0.84 (0.54,1.30)	1.23 (0.53,2.87)	1.78 (0.61,5.19)	0.58 (0.31,1.08)	0.15
CYP27A1						
Negative	Cases/controls	201/1029	61/315	37/195	103/519	
	OR (95% CI)^a^	0.81 (0.48,1.35)	1.08 (0.39,2.96)	1.68 (0.45,6.27)	0.54 (0.26,1.11)	0.25
Positive	Cases/controls	72/1029	22/315	14/195	36/519	
	OR (95% CI)^a^	1.31 (0.60,2.89)	2.01 (0.42,9.58)	2.30 (0.26,20.48)	1.07 (0.35,3.26)	0.79
*p*_het_		0.32	0.51	0.73	0.32	
CYP7B1						
Negative	Cases/controls	156/1029	46/315	30/195	80/519	
	OR (95% CI)^a^	0.90 (0.51,1.59)	1.54 (0.49,4.84)	5.79 (1.22,27.4)	0.58 (0.26,1.29)	0.15
Positive	Cases/controls	86/1029	29/315	19/195	38/519	
	OR (95% CI)^a^	0.74 (0.35,1.56)	1.10 (0.26,4.64)	0.53 (0.08,3.37)	0.37 (0.12,1.11)	0.17
*p*_het_		0.60	0.82	0.22	0.61	
LXR-β						
Negative	Cases/controls	122/1029	38/315	22/195	62/519	
	OR (95% CI)^a^	0.85 (0.46,1.59)	1.15 (0.33,4.03)	16.64 (2.54,108.87)	0.44 (0.18,1.09)	0.02
Positive	Cases/controls	164/1029	48/315	32/195	84/519	
	OR (95% CI)^a^	0.87 (0.50,1.53)	1.31 (0.43,4.01)	0.76 (0.18,3.14)	0.72 (0.33,1.58)	0.69
*p*_het_		0.91	0.70	0.02	0.34	
ERβ						
Negative	Cases/controls	83/1029	24/315	18/195	41/519	
	OR (95% CI)^a^	0.73 (0.35,1.53)	1.24 (0.29,5.37)	6.66 (0.97,45.7)	0.31 (0.10, 0.92)	0.06
Positive	Cases/controls	203/1029	62/315	36/195	105/519	
	OR (95% CI)^a^	0.86 (0.52,1.44)	1.27 (0.46,3.5)	0.93 (0.22,3.8)	0.72 (0.36,1.47)	0.66
*p*_het_^c^		0.67	0.96	0.17	0.12	

Missing: CYP7B1 = 44, CYP27A1 = 14. Also, 8 women have missing for date at blood draw and fasting status at blood collection: 1 case and 7 controls

^a^Odds ratios (ORs) per one-unit increase in log2 transformed 27HC; all ORs adjusted for matching factors plus age at first menstrual period, BMI, age at first full term pregnancy, number of full term pregnancies, and matching factors, namely, age, time, and date at blood draw, menopausal status, exogenous hormone use, and fasting status at blood collection, and additionally menstrual phase for premenopausal women

^b^Comparing pre-, peri-, postmenopausal

^c^Comparing tumor expression

## Discussion

Following our findings on circulating 27HC and breast cancer risk [8], this exploratory study provides novel data on associations between breast cancer case characteristics and epidemiologic factors and 27HC-related markers in breast tumor tissue, and is the first study on circulating 27HC and breast cancer risk by CYP27A1, CYP7B1, LXR-β, or ERβ tumor markers. We observed limited differences in the evaluated characteristics, and limited statistically significant heterogeneity in the association between circulating 27HC and breast cancer risk was observed by tumor tissue expression of the investigated markers.

The literature to date on the role of 27HC in the etiology of breast cancer is conflicting with potentially different roles for this oxysterol in risk and progression, for example experimental models suggested a growth-promotion role [6, 7], and epidemiologic studies showing an inverse association between circulating 27HC and breast cancer risk in postmenopausal women [8] and *CYP27A1* mRNA expression and death in breast cancer patients age 50 years and younger [10]. 27HC and circulating cholesterol are well correlated in our data (*r* = 0.45). On balance, prior studies do not support a strong association between circulating cholesterol and breast cancer risk [18] or survival [19, 20], with an inverse association reported between total and HDL cholesterol and risk in a meta-analysis [18] and postmenopausal women in the current study population [8]. Cholesterol-lowering drug use and breast cancer risk (predominantly statins) [21, 22] and survival [10, 23, 24] have been investigated with cholesterol-lowering medications, again mainly statins, proposed as a strategy to improve prognosis [24]. However, the association between statins and prognosis remains to be confirmed. Data on 27HC in the female breast is limited, though an analysis evaluating 27HC in the breast tissue of 40 breast cancer patients and 17 control women reported higher 27HC in the normal breast tissue of women with breast cancer (3x higher vs. controls) with elevated concentrations in the tumor tissue itself (6.9x higher than controls) [7].

CYP27A1 and CYP7B1 are expressed in both normal and malignant breast tissue, indicating capacity for local 27HC synthesis and catabolism. Differences in CYP27A1 and CYP7B1 in the normal breast as compared to malignant tissue are not well established. However, one study suggests similar expression of CYP27A1 but markedly different CYP7B1 in normal vs. breast tumor tissue. Specifically, CYP7B1 was 50% lower in ER+ tumor tissue relative to normal tissue [7] suggesting lower CYP7B1 may be responsible for the elevated 27HC observed in breast tumors. We investigated CYP27A1 and CYP7B1 protein expression in the tumor as markers indicative of local 27HC metabolism. Neither marker was associated with circulating 27HC, in line with previous findings on CYP27A1 [10] and a prior study reporting no association between tumor and circulating 27HC [7]. Tumor CYP27A1 protein expression was associated with lower circulating HDL concentrations, while tumor CYP7B1 was associated with lower triglycerides. In premenopausal women, tumor CYP27A1 protein expression was associated with higher circulating DHEAS concentrations, and in perimenopausal women, tumor CYP7B1 was associated with lower testosterone. To our knowledge, this has not previously been reported. One cell-line study suggested a role of CYP7B1 in androgenic signaling regulation [25] notably by converting androgen receptor ligands into less active metabolites. This association was also investigated in prostate cancer showing correlation between *CYP7B1* expression and androgen signaling activity [26, 27].

We observed no associations between CYP27A1 expression and breast cancer case characteristics, whereas CYP7B1-positive tumors were more likely to be PR-positive than CYP7B1-negative tumors. Kimbung et al. observed consistent differences in ER and nodal status, molecular subtype, and histologic grade by *CYP27A1* mRNA “low” vs. “high” [10]. Differences in grade, ER, and PR status have previously been reported using IHC [6]. We observed no significant heterogeneity in associations between circulating 27HC and breast cancer risk by CYP27A1 or CYP7B1 tumor expression though a statistically significant positive association between perimenopausal circulating 27HC and CYP7B1-negative breast cancer risk was observed.

The ERβ has been recognized for more than two decades; however, the clinical significance of the ERβ, in contrast to the clinically measured ERα, has not been established. 27HC causes conformational change in both the ERα and ERβ [2], and ERβ is known to be expressed in both ERα-positive and ERα-negative tumors [16] as observed in the current study (16.5% of ERβ-positive tumors were ERα/PR-negative). A higher proportion of ERβ-positive tumors were Bcl-2-low; prior studies have evaluted associations between tumor hormone receptor status and Bcl-2 expression, and expression of ERβ or Bcl-2 as prognostic markers [28–32]. The lack of association between other breast cancer characteristics observed in the current study is in line with previous studies [33, 34], though an association between ERβ expression and lower-tumor grade has been reported, as have significant associations between ERβ and ERα, between ERβ and PR, and between ERβ and HER2 expression (all *p* < 0.01) [16]. We observed no statistically significant heterogeneity in the association between circulating 27HC and breast cancer risk by ERβ expression, 27HC was only significantly associated with lower breast cancer risk among women postmenopausal at blood collection and negative for the ERβ. To our knowledge, this has not previously been described. In lung cancer, one prior study showed that treatment with 27HC increased cell proliferation in ERB-positive lung cancers [35]. No heterogeneity by ERα was observed in our previous investigation [8]. 27HC has been shown to exert effects beyond the ER (e.g., immune pathway [36], LXR [6, 7]).

In a previous experimental study, 27HC was shown to increase the transcriptional activity of LXR and thus was suggested to be an endogenous ligand for these receptors [37]. 27HC appears to increase metastases through the liver X receptor (LXR), and not the ER, notable given LXR agonists are generally associated with inhibition of breast cancer growth [38–40]. 27HC and LXR agonist GW3965 induced an increase in lung metastases, whereas estradiol had no effect [6]. This LXR-mediated increase in metastases appeared to be independent of the ER. We observed no heterogeneity in the associations between circulating 27HC and breast cancer by LXR-β status except among perimenopausal women where the risk of LXR-β-negative breast cancer was higher with higher circulating 27HC concentrations. These results are not in line with the above described literature, and we are unaware of any underlying biological explanation for this association. These results should be interpreted with caution given the limited sample size in this subgroup (*n* = 54 total perimenopausal cases) and wide confidence intervals associated with the ORs. Our study measured LXR-β expression rather than using a marker of LXR activity such as ABCA1 expression. LXR-α has also been implicated in breast cancer pathogenesis and in the 27HC-mediated response [36] and should therefore be considered in future studies.

No association with tumor makers was observed for reproductive and lifestyle factors in the current study. The literature is sparse regarding factors associated with circulating 27HC. Our cross-sectional study, which aimed to characterize the association between dietary, reproductive, lifestyle, and anthropometric factors and circulating 27HC in a sample of women without cancer [41], showed no or only a very modest impact of dietary habits, reproductive factors, and lifestyle factors on circulating 27HC concentrations.

27HC concentrations were measured in serum samples collected at study baseline, and repeat blood samples were not available; however, our prior study showed a high within-person reproducibility for circulating 27HC over 1 year [42]. We had tumor blocks available only for a subset of the cases, which may impact the generalizability of our findings to a broader population of breast cancer cases, given cases with tumor tissue available and included on the TMAs were younger at diagnosis, diagnosed with more advanced cancer (grade II or III) and ductal morphology (*p* < 0.001), compared to cases not included on the TMAs. Absolute differences in age at blood collection were relatively small (e.g., cases having TMAs available were, on average, 2.8 years younger than the cases not included in the analysis) and, while menopausal status has a weak, but statistically significant, impact on circulating 27HC (6.45% lower in premenopausal vs. postmenopausal women [41]), the proportion of postmenopausal women did not differ by TMA availability (with 51% and without 50% TMA), and the risk analyses were stratified by menopausal status. Thus, it is unlikely that differences in epidemiologic characteristics by TMA availability substantially impacted our results. An additional limitation is that the characterization of ERβ remains an issue due to its various isoforms [16] and the lack of specificity [43] of IHC assays. Thus, our results, as well as those in other studies using ERβ IHC antibodies, should be considered in light of the described issues with ERβ characterization using IHC. The distribution of positive/negative status for the tumor markers was comparable with distributions of ERβ [16, 33, 34], CYP27A1 [6], and CYP7B1 [15] previously reported. In the interpretation of our results, it should also be noted that preclinical studies implicate 27HC in breast cancer progression, while our epidemiological study evaluated circulating 27HC and breast cancer risk. Finally, we made many statistical comparisons in this investigation and did not adjust for multiple comparisons, thus we cannot rule out chance as an explanation for our statistically significant findings.

## Conclusion

This exploratory study is the first prospective human study investigating circulating 27HC and breast cancer risk by breast tissue tumor markers, and the first evaluation of 27HC-related tumor tissue markers and reproductive, anthropometric, and lifestyle factors. We observed limited associations between breast cancer case characteristics and the investigated tumor markers, and no significant heterogeneity in associations between circulating 27HC and breast cancer risk by breast tumor CYP27A1, CYP7B1, or ERβ expression, and limited heterogeneity by LXR-β. Larger-scale studies are required to confirm these findings.

## Supplementary information

Additional file 1: **Table S1.** Distribution of tumor characteristics, reproductive and lifestyle factors, lipid and hormonal biomarkers by tissue microarrays availability.

Additional file 2: Table S2. Association between breast tumor markers.

Additional file 3: Table S3. Reproductive and lifestyle factors by tumor marker status.

Additional file 4: Table S4. Geometric mean concentrations of Lipid and hormonal biomarkers in blood by tumor marker status, stratified by menopausal and HT user status.

## Abbreviations

27HC: 27-Hydroxycholeterol
BMI: Body mass index
CI: Confidence intervals
CYP27A1: Cholesterol 27-hydroxylase
CYP7B1: Oxysterol 7-alpha-hydroxylase
DHEAS: Dehydroepiandrosterone sulfate
EPIC: European Prospective Investigation into Cancer and Nutrition
ERβ: Estrogen receptor beta
FFPE: Formalin-fixed paraffin-embedded
FTP: Full-term pregnancy
HT: Hormone therapy
LC-MS: Liquid chromatography–mass spectrometry
LXR: Liver X receptor
OC: Oral contraceptive
OR: Odds ratios
QC: Quality control
TMA: Tissue microarray

## References

[1] Umetani M, Shaul PW (2011). 27-Hydroxycholesterol: the first identified endogenous SERM. Trends Endocrinol Metab.

[2] DuSell CD, McDonnell DP (2008). 27-Hydroxycholesterol: a potential endogenous regulator of estrogen receptor signaling. Trends Pharmacol Sci.

[3] Starkey NJE, Li Y, Drenkhahn-Weinaug SK, Liu J, Lubahn DB (2018). 27-Hydroxycholesterol is an estrogen receptor beta-selective negative allosteric modifier of 17beta-estradiol binding. Endocrinology.

[4] Leung YK, Lee MT, Lam HM, Tarapore P, Ho SM (2012). Estrogen receptor-beta and breast cancer: translating biology into clinical practice. Steroids.

[5] Umetani M, Domoto H, Gormley AK, Yuhanna IS, Cummins CL, Javitt NB, Korach KS, Shaul PW, Mangelsdorf DJ (2007). 27-Hydroxycholesterol is an endogenous SERM that inhibits the cardiovascular effects of estrogen. Nat Med.

[6] Nelson ER, Wardell SE, Jasper JS, Park S, Suchindran S, Howe MK, Carver NJ, Pillai RV, Sullivan PM, Sondhi V (2013). 27-Hydroxycholesterol links hypercholesterolemia and breast cancer pathophysiology. Science (New York, NY).

[7] Wu Q, Ishikawa T, Sirianni R, Tang H, McDonald JG, Yuhanna IS, Thompson B, Girard L, Mineo C, Brekken RA (2013). 27-Hydroxycholesterol promotes cell-autonomous, ER-positive breast cancer growth. CellRep.

[8] Lu DL, Le Cornet C, Sookthai D, Johnson TS, Kaaks R, Fortner RT (2019). Circulating 27-hydroxycholesterol and breast cancer risk: results from the EPIC-Heidelberg cohort. J Natl Cancer Inst.

[9] Dalenc F, Iuliano L, Filleron T, Zerbinati C, Voisin M, Arellano C, Chatelut E, Marquet P, Samadi M, Roche H (2017). Circulating oxysterol metabolites as potential new surrogate markers in patients with hormone receptor-positive breast cancer: results of the OXYTAM study. J Steroid Biochem Mol Biol.

[10] Kimbung S, Chang C, Bendahl PO, Dubois L, Thompson WJ, McDonnell DP, Borgquist S (2017). Impact of 27-hydroxylase (CYP27A1) and 27-hydroxycholesterol in breast cancer. Endocr Relat Cancer.

[11] Revilla G, Pons MP, Baila-Rueda L, Garcia-Leon A, Santos D, Cenarro A, Magalhaes M, Blanco RM, Moral A, Ignacio Perez J (2019). Cholesterol and 27-hydroxycholesterol promote thyroid carcinoma aggressiveness. Sci Rep.

[12] Riboli E, Hunt KJ, Slimani N, Ferrari P, Norat T, Fahey M, Charrondiere UR, Hemon B, Casagrande C, Vignat J (2002). European prospective investigation into cancer and nutrition (EPIC): study populations and data collection. Public Health Nutr.

[13] Boeing H, Wahrendorf J, Becker N (1999). EPIC-Germany--A source for studies into diet and risk of chronic diseases. European Investigation into Cancer and Nutrition. Ann Nutr Metab.

[14] Nattenmuller CJ, Kriegsmann M, Sookthai D, Fortner RT, Steffen A, Walter B, Johnson T, Kneisel J, Katzke V, Bergmann M (2018). Obesity as risk factor for subtypes of breast cancer: results from a prospective cohort study. BMC Cancer.

[15] Pu H, Zhang Q, Zhao C, Shi L, Wang Y, Wang J, Zhang M (2015). Overexpression of G6PD is associated with high risks of recurrent metastasis and poor progression-free survival in primary breast carcinoma. World J Surg Oncol.

[16] Marotti JD, Collins LC, Hu R, Tamimi RM (2010). Estrogen receptor-beta expression in invasive breast cancer in relation to molecular phenotype: results from the nurses&apos; Health Study. Modern Pathol.

[17] Wang M, Spiegelman D, Kuchiba A, Lochhead P, Kim S, Chan AT, Poole EM, Tamimi R, Tworoger SS, Giovannucci E (2016). Statistical methods for studying disease subtype heterogeneity. Stat Med.

[18] Touvier M, Fassier P, His M, Norat T, Chan DS, Blacher J, Hercberg S, Galan P, Druesne-Pecollo N, Latino-Martel P (2015). Cholesterol and breast cancer risk: a systematic review and meta-analysis of prospective studies. Br J Nutr.

[19] His M, Dartois L, Fagherazzi G, Boutten A, Dupré T, Mesrine S, Boutron-Ruault M-C, Clavel-Chapelon F, Dossus L (2016). Associations between serum lipids and breast cancer incidence and survival in the E3N prospective cohort study. Cancer Causes Control.

[20] Wulaningsih W, Vahdaninia M, Rowley M, Holmberg L, Garmo H, Malmstrom H, Lambe M, Hammar N, Walldius G, Jungner I, et al. Prediagnostic serum glucose and lipids in relation to survival in breast cancer patients: a competing risk analysis. BMC Cancer. 2015;15(1) 10.1186/s12885-015-1928-z PMC4650114 26577580

[21] Eliassen AH, Colditz GA, Rosner B, Willett WC, Hankinson SE (2005). Serum lipids, lipid-lowering drugs, and the risk of breast cancer. Arch Intern Med.

[22] Borgquist S, Tamimi RM, Chen WY, Garber JE, Eliassen AH, Ahern TP (2016). Statin use and breast cancer risk in the nurses’ health study. Cancer Epidemiol Biomarkers Prev.

[23] Nickels S, Vrieling A, Seibold P, Heinz J, Obi N, Flesch-Janys D, Chang-Claude J (2013). Mortality and recurrence risk in relation to the use of lipid-lowering drugs in a prospective breast cancer patient cohort. PLoS One.

[24] Ahern TP, Lash TL, Damkier P, Christiansen PM, Cronin-Fenton DP (2014). Statins and breast cancer prognosis: evidence and opportunities. Lancet Oncol.

[25] Lundqvist J, Norlin M (2012). Effects of CYP7B1-related steroids on androgen receptor activation in different cell lines. Biochim Biophys Acta.

[26] Lutz SZ, Hennenlotter J, Scharpf MO, Sailer C, Fritsche L, Schmid V, Kantartzis K, Wagner R, Lehmann R, Berti L (2018). Androgen receptor overexpression in prostate cancer in type 2 diabetes. Mol Metab.

[27] Tang W, Norlin M (2006). Regulation of steroid hydroxylase CYP7B1 by androgens and estrogens in prostate cancer LNCaP cells. Biochem Biophys Res Commun.

[28] Honma N, Horii R, Ito Y, Saji S, Younes M, Iwase T, Akiyama F (2015). Differences in clinical importance of Bcl-2 in breast cancer according to hormone receptors status or adjuvant endocrine therapy. BMC Cancer.

[29] Callagy GM, Pharoah PD, Pinder SE, Hsu FD, Nielsen TO, Ragaz J, Ellis IO, Huntsman D, Caldas C (2006). Bcl-2 is a prognostic marker in breast cancer independently of the Nottingham prognostic index. Clin Cancer Res.

[30] Hwang KT, Woo JW, Shin HC, Kim HS, Ahn SK, Moon HG, Han W, Park IA, Noh DY (2012). Prognostic influence of BCL2 expression in breast cancer. Int J Cancer.

[31] Dawson SJ, Makretsov N, Blows FM, Driver KE, Provenzano E, Le Quesne J, Baglietto L, Severi G, Giles GG, McLean CA (2010). BCL2 in breast cancer: a favourable prognostic marker across molecular subtypes and independent of adjuvant therapy received. Br J Cancer.

[32] Tan W, Li Q, Chen K, Su F, Song E, Gong C (2016). Estrogen receptor beta as a prognostic factor in breast cancer patients: a systematic review and meta-analysis. Oncotarget.

[33] Hopp TA, Weiss HL, Parra IS, Cui Y, Osborne CK, Fuqua SA (2004). Low levels of estrogen receptor beta protein predict resistance to tamoxifen therapy in breast cancer. Clin Cancer Res.

[34] Gruvberger-Saal SK, Bendahl PO, Saal LH, Laakso M, Hegardt C, Eden P, Peterson C, Malmstrom P, Isola J, Borg A (2007). Estrogen receptor expression is associated with tamoxifen response in ER -negative breast carcinoma. Clin Cancer Res.

[35] Hiramitsu S, Ishikawa T, Lee WR, Khan T, Crumbley C, Khwaja N, Zamanian F, Asghari A, Sen M, Zhang Y (2018). Estrogen receptor beta-mediated modulation of lung cancer cell proliferation by 27-hydroxycholesterol. Front Endocrinol (Lausanne).

[36] Baek AE, Yu YA, He S, Wardell SE, Chang CY, Kwon S, Pillai RV, McDowell HB, Thompson JW, Dubois LG (2017). The cholesterol metabolite 27 hydroxycholesterol facilitates breast cancer metastasis through its actions on immune cells. Nat Commun.

[37] DuSell CD, Nelson ER, Wang X, Abdo J, Modder UI, Umetani M, Gesty-Palmer D, Javitt NB, Khosla S, McDonnell DP (2010). The endogenous selective estrogen receptor modulator 27-hydroxycholesterol is a negative regulator of bone homeostasis. Endocrinology.

[38] Vedin L-L, Lewandowski SA, Parini P, Gustafsson J-Å, Steffensen KR (2009). The oxysterol receptor LXR inhibits proliferation of human breast cancer cells. Carcinogenesis.

[39] Nguyen-Vu T, Vedin L-L, Liu K, Jonsson P, Lin JZ, Candelaria NR, Candelaria LP, Addanki S, Williams C, Gustafsson J-Å (2013). Liver × receptor ligands disrupt breast cancer cell proliferation through an E2F-mediated mechanism. Breast Cancer Res.

[40] El Roz A, Bard J-M, Huvelin J-M, Nazih H (2012). LXR agonists and ABCG1-dependent cholesterol efflux in MCF-7 breast cancer cells: relation to proliferation and apoptosis. Anticancer Res.

[41] Le Cornet C, Johnson TS, Lu DL, Kaaks R, Fortner RT (2020). Association Between Lifestyle, Dietary, Reproductive, and Anthropometric Factors and Circulating 27-hydroxycholesterol in EPICHeidelberg. Cancer Causes Control.

[42] Lu DL, Sookthai D, Le Cornet C, Katzke VA, Johnson TS, Kaaks R, Fortner RT (2017). Reproducibility of serum oxysterols and lanosterol among postmenopausal women: Results from EPIC-Heidelberg. Clin Biochem.

[43] Nelson AW, Groen AJ, Miller JL, Warren AY, Holmes KA, Tarulli GA, Tilley WD, Katzenellenbogen BS, Hawse JR, Gnanapragasam VJ (2017). Comprehensive assessment of estrogen receptor beta antibodies in cancer cell line models and tissue reveals critical limitations in reagent specificity. Mol Cell Endocrinol.

