# Supplementary material for: Circulating 27-hydroxycholesterol and breast cancer tissue expression of CYP27A1, CYP7B1, LXR-β, and ERβ: results from the EPIC-Heidelberg cohort
Source: Breast Cancer Res. 2020 Feb 19;22:23. doi: 10.1186/s13058-020-1253-6 (PMC7031866; doi:10.1186/s13058-020-1253-6)
Supplement: Supplementary file 1 — Table S1. Distribution of tumor characteristics, reproductive and lifestyle factors, lipid and hormonal biomarkers by tissue microarrays availability. [file 13058_2020_1253_MOESM1_ESM.docx]

**Supplementary table 1: Distribution of tumor characteristics, reproductive and lifestyle factors, lipid and hormonal biomarkers by tissue microarrays availability**

|  | Tissue microarrays assessment available | | |
| --- | --- | --- | --- |
|  | No ^a^ | Yes^b^ | p-value^c^ |
| N | 243 | 287 |  |
| Age at diagnosis | 61.9 ± 8.54 | 59.1 ± 8.03 | <0.01 |
| Age at recruitment | 51.09 ± 7.88 | 51.40 ± 7.91 | 0.66 |
| Receptor subtype |  |  |  |
| ER+ | 92 (77.3) | 236 (82.5) | 0.27 |
| ER- | 27 (22.7) | 50 (17.5) |  |
| ER+/PR+ | 81 (75.7) | 206 (82.7) | 0.14 |
| ER-/PR- | 26 (24.3) | 43 (17.3) |  |
| PR+ | 82 (68.9) | 213 (74.5) | 0.27 |
| PR- | 37 (31.1) | 73 (25.5) |  |
| HER2+ | 17 (14.7) | 46 (16.1) | 0.76 |
| HER2- | 99 (85.3) | 240 (83.9) |  |
| ER-/PR-/HER2- | 16 (13.8) | 31 (10.8) | 0.40 |
| ER+or PR+or HER2+ | 100 (86.2) | 255 (89.2) |  |
| Ki67 |  |  |  |
| Low | 5 (2.1) | 46 (16.0) | 0.11 |
| High | 53 (21.8) | 219 (76.3) |  |
| bcl2 |  |  |  |
| Low | 44 (18.1) | 165 (57.5) | 0.07 |
| High | 15 (6.2) | 101 (35.2) |  |
| p53 |  |  |  |
| Low | 15 (6.2) | 52 (18.1) | 0.38 |
| High | 45 (18.5) | 214 (74.6) |  |
| Breast cancer stage |  |  |  |
| Local | 133 (67.5) | 175 (62.3) | 0.33 |
| Regional | 58 (29.4) | 100 (35.6) |  |
| Metastasis | 6 (3.0) | 6 (2.1) |  |
| Breast cancer grade |  |  |  |
| Grade I | 49 (24.3) | 38 (13.3) | <0.01 |
| Grade II | 103 (51.0) | 163 (57.2) |  |
| Grade III | 50 (24.8) | 84 (29.5) |  |
| Breast cancer morphology |  |  |  |
| Ductal carcinoma or Infiltrating duct carcinoma | 127 (52.3) | 202 (70.4) | <0.01 |
| Lobular carcinoma | 56 (23.0) | 56 (19.5) |  |
| Other | 60 (24.7) | 29 (10.1) |  |
| Cambridge physical activity index |  |  |  |
| Inactive | 38 (15.6) | 27 (9.4) | 0.13 |
| Moderately inactive | 79 (32.5) | 111 (38.7) |  |
| Moderately active | 71 (29.2) | 87 (30.3) |  |
| Active | 55 (22.6) | 62 (21.6) |  |
| BMI |  |  |  |
| Normal | 130 (53.5) | 161 (56.1) | 0.79 |
| Overweight | 81 (33.3) | 88 (30.7) |  |
| Obese | 32 (13.2) | 38 (13.2) |  |
| Full term pregnancy |  |  |  |
| No | 48 (19.8) | 56 (19.6) | 1.00 |
| Yes | 195 (80.2) | 230 (80.4) |  |
| Number of full term pregnancy |  |  |  |
| 0 | 48 (19.8) | 56 (19.5) | 0.92 |
| 1 | 59 (24.3) | 66 (23.0) |  |
| 2 | 100 (41.2) | 116 (40.4) |  |
| 3+ | 36 (14.8) | 49 (17.1) |  |
| Use hormone |  |  |  |
| No | 135 (55.6) | 174 (60.6) | 0.25 |
| Yes | 108 (44.4) | 113 (39.4) |  |
|  |  |  |  |
| Hormone use duration | 6.14 ± 5.10 | 5.80 ± 4.55 | 0.59 |
| Menopausal status |  |  |  |
| Premenopausal | 76 (31.3) | 86 (30.0) | 0.95 |
| Postmenopausal | 122 (50.2) | 147 (51.2) |  |
| Perimenopausal | 45 (18.5) | 54 (18.8) |  |
| Breastfeeding |  |  |  |
| No | 90 (37.0) | 100 (35.2) | 0.72 |
| Yes | 153 (63.0) | 184 (64.8) |  |
| Smoking status |  |  |  |
| Never | 126 (51.9) | 157 (54.7) | 0.24 |
| Former | 77 (31.7) | 72 (25.1) |  |
| Smoker | 40 (16.5) | 57 (19.9) |  |
| Alcohol consumption lifetime | 9.23 ± 12.53 | 7.40 ± 9.23 | 0.06 |
| Lipid biomarkers |  |  |  |
| 27-Hydroxycholesterol (nM) | 194.7 (189.6-200) | 192.4 (187.7-197.2) | 0.51 |
| Cholesterol (mg/dl) | 233.2 (228.3-238.3) | 236.4 (231.8-241.1) | 0.36 |
| Triglycerides (mg/dl) | 115.6 (108.5-123.1) | 116.4 (109.9-123.4) | 0.87 |
| High-density lipoproteins (mg/dl) | 68.3 (66.2-70.5) | 67.4 (65.5-69.3) | 0.51 |
| Low-density lipoproteins (mg/dl) | 131.9 (127.4-136.5) | 136.3 (132-140.7) | 0.17 |
| Endogenous sex steroid hormone concentrations |  |  |  |
| Testosterone (ng/ml) | 0.3 (0.3-0.4) | 0.3 (0.3-0.4) | 0.69 |
| Progesterone (ng/ml) | 7.5 (6.7-8.5) | 7.6 (6.9-8.5) | 0.87 |
| Estrone (pg/ml) | 41.7 (35.9-48.3) | 39.2 (34.2-44.9) | 0.54 |
| Estradiol (pg/ml) | 75.2 (64.8-87.2) | 75.1 (65.6-85.8) | 0.99 |
| DHEAS (µg/dl) | 100.6 (90.2-112.2) | 104.2 (94.3-115.2) | 0.64 |

^a^Missing for cases without tissue microarrays: ER=124, PR=124, HER2=127, Breast cancer stage=46, Breast cancer grade=41, Ki67=185, bcl2=184, p53=183, Hormone use duration among hormone users=6, Cholesterol=1, Triglyceride=1, HDL=1, LDL=1, testosterone=6, progesterone=5, estrone=15, DHEAS=4.

^b^Missing among cases with tissue microarrays: ER=1, PR=1, HER2=1, breast cancer stage=6, breast cancer grade=2, Ki67=22, bcl2=21, p53=21, Full term pregnancy=1, Hormone use duration among Hormone users=7, Smoking status=1, testosterone=5, progesterone=5, estrone=8

^c^Fisher’s exact tests for categorical variable [n (%)] or Welch's t-test for continuous variable [mean± std]
