# Supplementary material for: Circulating 27-hydroxycholesterol and breast cancer tissue expression of CYP27A1, CYP7B1, LXR-β, and ERβ: results from the EPIC-Heidelberg cohort
Source: Breast Cancer Res. 2020 Feb 19;22:23. doi: 10.1186/s13058-020-1253-6 (PMC7031866; doi:10.1186/s13058-020-1253-6)
Supplement: Supplementary file 2 — Association between breast tumor markers. [file 13058_2020_1253_MOESM2_ESM.docx]

**Supplementary table 2: Association between breast tumor markers**

|  | **CYP27A1** | | | **CYP7B1** | | | **LXR-β** | | |
| --- | --- | --- | --- | --- | --- | --- | --- | --- | --- |
|  | Negative | Positive | p^a^ | Negative | Positive | p^a^ | Negative | Positive | p^a^ |
| **CYP27A1** | | | | | | | | | |
| Negative | -- | -- | -- | -- | -- | -- | -- | -- | -- |
| Positive | -- | -- | -- | -- | -- | -- | -- | -- | -- |
| **CYP7B1** | | | | | | | | | |
| Negative | 108 (62) | 36 (63) |  | -- | -- | -- | -- | -- | -- |
| Positive | 65 (38) | 21 (37) | 0.99 | -- | -- | -- | -- | -- | -- |
| **LXR-β** | | | | | | | | | |
| Negative | 87 (43) | 24 (33) |  | 74 (47) | 36 (42) |  | -- | -- | -- |
| Positive | 114 (57) | 48 (67) | 0.16 | 83 (53) | 50 (58) | 0.50 | -- | -- | -- |
| **ERβ** | | | | | | | | | |
| Negative | 61 (30) | 10 (14) |  | 54 (34) | 23 (27) |  | 48 (39) | 36 (22) |  |
| Positive | 140 (70) | 62 (86) | <0.01 | 103 (66) | 63 (73) | 0.25 | 75 (61) | 128 (78) | <0.01 |

^a^two-tailed Fisher's exact test [n (%)]
