# Supplementary material for: Circulating 27-hydroxycholesterol and breast cancer tissue expression of CYP27A1, CYP7B1, LXR-β, and ERβ: results from the EPIC-Heidelberg cohort
Source: Breast Cancer Res. 2020 Feb 19;22:23. doi: 10.1186/s13058-020-1253-6 (PMC7031866; doi:10.1186/s13058-020-1253-6)
Supplement: Supplementary file 3 — Reproductive and lifestyle factors by tumor marker status. [file 13058_2020_1253_MOESM3_ESM.docx]

**Supplemental Table 3: Reproductive and lifestyle factors by tumor marker status.**

|  | CYP27A1 | | | CYP7B1 | | | LXR-β | | | ERβ | | |
| --- | --- | --- | --- | --- | --- | --- | --- | --- | --- | --- | --- | --- |
|  | Negative | Positive | p^a^ | Negative | Positive | p^a^ | Negative | Positive | p^a^ | Negative | Positive | p^a^ |
| N | 201 | 72 |  | 157 | 86 |  | 123 | 164 |  | 84 | 203 |  |
| Age at recruitment | 51.0 ± 8.0 | 52.2 ± 7.7 | 0.26 | 51.3 ± 8.1 | 50.6 ± 7.8 | 0.28 | 51.3 ± 8.3 | 51.4 ± 7.6 | 0.91 | 50.8 ± 7.5 | 51.6 ± 8.1 | 0.43 |
| Cambridge physical activity index | |  |  |  |  |  |  |  |  |  |  |  |
| Inactive | 23 (11.4) | 4 (5.6) | 0.17 | 13 (8.3) | 6 (7.0) | 0.71 | 8 (6.5) | 19 (11.6) | 0.49 | 10 (11.9) | 17 (8.4) | 0.69 |
| Moderately inactive | 84 (41.8) | 24 (33.3) |  | 65 (41.4) | 31 (36.0) |  | 47 (38.2) | 64 (39.0) |  | 29 (34.5) | 82 (40.4) |  |
| Moderately active | 56 (27.9) | 25 (34.7) |  | 44 (28.0) | 30 (34.9) |  | 39 (31.7) | 48 (29.3) |  | 26 (31.0) | 61 (30.0) |  |
| Active | 38 (18.9) | 19 (26.4) |  | 35 (22.3) | 19 (22.1) |  | 29 (23.6) | 33 (20.1) |  | 19 (22.6) | 43 (21.2) |  |
| Body mass index |  |  |  |  |  |  |  |  |  |  |  |  |
| Continuous | 25.0 ± 4.1 | 25.8 ± 4.9 | 0.24 | 25.6 ± 4.9 | 24.6 ± 3.9 | 0.07 | 25.4 ± 4.7 | 25.2 ± 4.5 | 0.67 | 25.8 ± 5.1 | 25.1 ± 4.3 | 0.31 |
| Normal | 113 (56.2) | 39 (54.2) | 0.10 | 86 (54.8) | 54 (62.8) | 0.46 | 66 (53.7) | 95 (57.9) | 0.70 | 46 (54.8) | 115 (56.7) | 0.78 |
| Overweight | 68 (33.8) | 19 (26.4) |  | 49 (31.2) | 23 (26.7) |  | 41 (33.3) | 47 (28.7) |  | 25 (29.8) | 63 (31.0) |  |
| Obese | 20 (10.0) | 14 (19.4) |  | 22 (14.0) | 9 (10.5) |  | 16 (13.0) | 22 (13.4) |  | 13 (15.5) | 25 (12.3) |  |
| Full term pregnancy |  |  |  |  |  |  |  |  |  |  |  |  |
| No | 39 (19.5) | 16 (22.2) | 0.61 | 30 (19.2) | 18 (20.9) | 0.75 | 25 (20.5) | 31 (18.9) | 0.76 | 21 (25.0) | 35 (17.3) | 0.14 |
| Yes | 161 (80.5) | 56 (77.8) |  | 126 (80.8) | 68 (79.1) |  | 97 (79.5) | 133 (81.1) |  | 63 (75.0) | 167 (82.7) |  |
| Number of full term pregnancy | |  |  |  |  |  |  |  |  |  |  |  |
| 0 | 39 (19.4) | 16 (22.2) | 0.19 | 30 (19.1) | 18 (20.9) | 0.78 | 25 (20.3) | 31 (18.9) | 0.10 | 21 (25.0) | 35 (17.2) | 0.33 |
| 1 | 48 (23.9) | 13 (18.1) |  | 39 (24.8) | 19 (22.1) |  | 20 (16.3) | 46 (28.0) |  | 21 (25.0) | 45 (22.2) |  |
| 2 | 75 (37.3) | 35 (48.6) |  | 64 (40.8) | 32 (37.2) |  | 57 (46.3) | 59 (36.0) |  | 31 (36.9) | 85 (41.9) |  |
| 3+ | 39 (19.4) | 8 (11.1) |  | 24 (15.3) | 17 (19.8) |  | 21 (17.1) | 28 (17.1) |  | 11 (13.1) | 38 (18.7) |  |
| Hormone user |  |  |  |  |  |  |  |  |  |  |  |  |
| No | 120 (59.7) | 45 (62.5) | 0.78 | 96 (61.1) | 55 (64.0) | 0.67 | 69 (56.1) | 105 (64.0) | 0.18 | 50 (59.5) | 124 (61.1) | 0.90 |
| Yes | 81 (40.3) | 27 (37.5) |  | 61 (38.9) | 31 (36.0) |  | 54 (43.9) | 59 (36.0) |  | 34 (40.5) | 79 (38.9) |  |
| Hormone use duration | 6.0 ± 4.6 | 5.6 ± 4.8 | 0.67 | 6.2 ± 4.9 | 5.1 ± 3.1 | 0.18 | 6.5 ± 5.0 | 5.2 ± 4.0 | 0.11 | 5.4 ± 4.4 | 6.0 ± 4.6 | 0.52 |
| Menopausal status |  |  |  |  |  |  |  |  |  |  |  |  |
| Premenopausal | 61 (30.3) | 22 (30.6) | 0.98 | 46 (29.3) | 29 (33.7) | 0.54 | 38 (30.9) | 48 (29.3) | 0.92 | 24 (28.6) | 62 (30.5) | 0.78 |
| Postmenopausal | 103 (51.2) | 36 (50.0) |  | 81 (51.6) | 38 (44.2) |  | 63 (51.2) | 84 (51.2) |  | 42 (50.0) | 105 (51.7) |  |
| Perimenopausal | 37 (18.4) | 14 (19.4) |  | 30 (19.1) | 19 (22.1) |  | 22 (17.9) | 32 (19.5) |  | 18 (21.4) | 36 (17.7) |  |
| Breastfeeding |  |  |  |  |  |  |  |  |  |  |  |  |
| No | 68 (34.2) | 27 (37.5) | 0.67 | 58 (37.7) | 28 (32.6) | 0.43 | 44 (36.7) | 56 (34.1) | 0.70 | 36 (42.9) | 64 (32.0) | 0.10 |
| Yes | 131 (65.8) | 45 (62.5) |  | 96 (62.3) | 58 (67.4) |  | 76 (63.3) | 108 (65.9) |  | 48 (57.1) | 136 (68.0) |  |
| Smoking status |  |  |  |  |  |  |  |  |  |  |  |  |
| Never | 111 (55.5) | 36 (50.0) | 0.73 | 84 (53.5) | 46 (53.5) | 0.19 | 61 (50.0) | 96 (58.5) | 0.18 | 43 (51.2) | 114 (56.4) | 0.55 |
| Former | 48 (24) | 21 (29.2) |  | 36 (22.9) | 28 (32.6) |  | 37 (30.3) | 35 (21.3) |  | 20 (23.8) | 52 (25.7) |  |
| Smoker | 41 (20.5) | 15 (20.8) |  | 36 (22.9) | 12 (14.0) |  | 24 (19.7) | 33 (20.1) |  | 21 (25.0) | 36 (17.8) |  |
| Alcohol consumption (g/d) ggglifetime | 7.5 ± 9.4 | 7.5 ± 9.5 | 0.98 | 8.2 ± 11.0 | 6.6 ± 5.7 | 0.14 | 7.3 ± 8.0 | 7.5 ± 10.1 | 0.81 | 7.3 ± 8.3 | 7.4 ± 9.6 | 0.93 |

^a^Fisher’s exact tests for categorical variable [n (%)] or Welch's t-test for continuous variable [mean± std];

Missing: full term pregnancy=1, hormone use duration among hormone users=7, smoking status=1
