# Supplementary material for: Circulating 27-hydroxycholesterol and breast cancer tissue expression of CYP27A1, CYP7B1, LXR-β, and ERβ: results from the EPIC-Heidelberg cohort
Source: Breast Cancer Res. 2020 Feb 19;22:23. doi: 10.1186/s13058-020-1253-6 (PMC7031866; doi:10.1186/s13058-020-1253-6)
Supplement: Supplementary file 4 — Geometric mean concentrations of Lipid and hormonal biomarkers in blood by tumor marker status, stratified by menopausal and HT user status. [file 13058_2020_1253_MOESM4_ESM.docx]

**Supplemental Table 4: Geometric mean concentrations of Lipid and hormonal biomarkers in blood by tumor marker status, stratified by menopausal and HT user status**

|  | CYP27A1 |  |  | CYP7B1 |  |  | LXR-β |  |  | ERβ |  |  |
| --- | --- | --- | --- | --- | --- | --- | --- | --- | --- | --- | --- | --- |
|  | Negative | Positive | p^a^ | Negative | Positive | p^a^ | Negative | Positive | p^a^ | Negative | Positive | p^a^ |
|  | 201 | 72 |  | 157 | 86 |  | 123 | 164 |  | 84 | 203 |  |
| **Overall** |  |  |  |  |  |  |  |  |  |  |  |  |
| 27-Hydroxycholesterol (nM) | 191.2 ± 1.2 | 197.8 ± 1.2 | 0.24 | 193.6 ± 1.2 | 187.8 ± 1.2 | 0.29 | 191.6 ± 1.2 | 193.0 ± 1.2 | 0.78 | 191.7 ± 1.3 | 192.7 ± 1.2 | 0.86 |
| Cholesterol (mg/dL) | 236.2 ± 1.2 | 239.2 ± 1.2 | 0.58 | 238.0 ± 1.2 | 229.8 ± 1.2 | 0.13 | 235.5 ± 1.2 | 237.0 ± 1.2 | 0.76 | 235.0 ± 1.2 | 236.9 ± 1.2 | 0.71 |
| Triglycerides | 112.0 ± 1.6 | 127.9 ± 1.7 | 0.06 | 123.1 ± 1.6 | 106.4 ± 1.5 | 0.02 | 112.8 ± 1.6 | 119.2 ± 1.7 | 0.34 | 121.8 ± 1.7 | 114.3 ± 1.6 | 0.34 |
| High-density lipoproteins | 68.8 ± 1.3 | 64.3 ± 1.3 | 0.04 | 67.4 ± 1.3 | 67.4 ± 1.2 | 1.00 | 67.7 ± 1.3 | 67.1 ± 1.3 | 0.74 | 64.9 ± 1.3 | 68.4 ± 1.3 | 0.10 |
| Low-density lipoproteins | 134.6 ± 1.3 | 142.4 ± 1.3 | 0.12 | 136.7 ± 1.3 | 131.7 ± 1.3 | 0.31 | 135.0 ± 1.3 | 137.2 ± 1.3 | 0.62 | 136.5 ± 1.3 | 136.1 ± 1.3 | 0.94 |
| **Premenopausal** |  |  |  |  |  |  |  |  |  |  |  |  |
| Testosterone | 0.4 ± 1.8 | 0.3 ± 1.4 | 0.48 | 0.36 ± 1.6 | 0.34 ± 1.5 | 0.68 | 0.4 ± 1.6 | 0.4 ± 1.7 | 0.97 | 0.4 ± 1.6 | 0.4 ± 1.7 | 0.97 |
| Progesterone^b^ | 4.6 ± 2.5 | 3.4 ± 2.8 | 0.26 | 4.1 ± 2.8 | 4.11 ± 2.4 | 0.99 | 4.7 ± 2.7 | 4.0 ± 2.5 | 0.48 | 5.0 ± 2.5 | 4.1 ± 2.6 | 0.42 |
| Estrone^b^ | 58.8 ± 1.6 | 50.2 ± 1.3 | 0.05 | 56.8 ± 1.6 | 56.0 ± 1.4 | 0.88 | 54.2 ± 1.5 | 58.1 ± 1.5 | 0.44 | 56.7 ± 1.4 | 56.1 ± 1.5 | 0.90 |
| Estradiol^b^ | 48.8 ± 2.7 | 35.1 ± 1.8 | 0.08 | 39.0 ± 2.9 | 61.8 ± 1.6 | 0.02 | 49.9 ± 2.5 | 40.9 ± 2.4 | 0.33 | 54.0 ± 1.9 | 41.6 ± 2.7 | 0.17 |
| DHEAS | 130.3 ± 2.2 | 169.4 ± 1.4 | 0.04 | 142.4 ± 2.3 | 139.3 ± 1.7 | 0.89 | 145.0 ± 1.7 | 136.5 ± 2.2 | 0.67 | 141.5 ± 1.6 | 139.8 ± 2.1 | 0.93 |
| **Perimenopausal** |  |  |  |  |  |  |  |  |  |  |  |  |
| Testosterone | 0.3 ± 2.1 | 0. 4 ± 2.0 | 0.52 | 0.40 ± 1.9 | 0.25 ± 2.1 | 0.03 | 0.4 ± 1.9 | 0.3 ± 2.1 | 0.83 | 0.4 ± 2.2 | 0.3 ± 1.9 | 0.42 |
| Progesterone | 8.2 ± 2.0 | 7.6 ± 2.0 | 0.74 | 8.7 ± 2.0 | 8.5 ± 1.8 | 0.91 | 8.0 ± 2.2 | 8.1 ± 1.8 | 0.94 | 6.5 ± 1.5 | 9.0 ± 2.2 | 0.05 |
| Estrone | 58.5 ± 3.7 | 59.5 ± 2.2 | 0.96 | 58.7 ± 2.0 | 57.0 ± 4.9 | 0.94 | 66.0 ± 2.1 | 53.2 ± 3.9 | 0.46 | 62.7 ± 5.6 | 55.9 ± 2.1 | 0.79 |
| Estradiol | 28.0 ± 4.0 | 26.2 ± 3.5 | 0.88 | 27.0 ± 4.0 | 27.1 ± 4.0 | 0.99 | 26.2 ± 4.5 | 26.4 ± 3.4 | 0.98 | 27.3 ± 3.3 | 25.8 ± 4.0 | 0.88 |
| DHEAS | 125.9 ± 1.8 | 98.0 ± 2.3 | 0.32 | 115.9 ± 2.0 | 119.8 ± 1.8 | 0.86 | 143.2 ± 1.8 | 105.1 ± 1.9 | 0.07 | 115.0 ± 2.2 | 121.3 ± 1.8 | 0.80 |
| **Postmenopausal, not using HT** |  |  |  |  |  |  |  |  |  |  |  |  |
| Testosterone | 0.3 ± 1.8 | 0.4 ± 1.5 | 0.40 | 0.34 ± 1.8 | 0.31 ± 1.3 | 0.60 | 0.4 ± 2.1 | 0.3 ± 1.5 | 0.65 | 0.3 ± 1.7 | 0.4 ± 1.7 | 0.57 |
| Progesterone | 7.5 ± 1.7 | 7.8 ± 1.6 | 0.83 | 7.5 ± 1.8 | 7.6 ± 1.3 | 0.93 | 8.6 ± 2.0 | 7.1 ± 1.5 | 0.31 | 7.3 ± 1.9 | 7.6 ± 1.6 | 0.82 |
| Estrone | 18.7 ± 1.7 | 27.7 ± 2.0 | 0.07 | 22.9 ± 1.8 | 24.0 ± 2.1 | 0.86 | 20.8 ± 1.5 | 22.2 ± 1.9 | 0.70 | 22.8 ± 2.0 | 21.3 ± 1.7 | 0.75 |
| Estradiol | 23.1 ± 3.2 | 14.7 ± 2.8 | 0.19 | 18.1 ± 3.6 | 17.9 ± 3.01 | 0.98 | 27.9 ± 3.4 | 17.2 ± 2.8 | 0.19 | 21.4 ± 5.3 | 19.8 ± 2.3 | 0.88 |
| DHEAS | 87.3 ± 1.9 | 85.2 ± 1.7 | 0.89 | 82.8 ± 1.8 | 78.7 ± 1.9 | 0.83 | 73.5 ± 1.8 | 89.6 ± 1.8 | 0.28 | 65.0 ± 2.0 | 93.1 ± 1.7 | 0.09 |
| **Postmenopausal, using HT** |  |  |  |  |  |  |  |  |  |  |  |  |
| Testosterone | 0.3 ± 2.4 | 0.3 ± 1.5 | 0.48 | 0.30 ± 2.6 | 0.28 ± 1.6 | 0.81 | 0.3 ± 1.5 | 0.3 ± 2.7 | 0.78 | 0.3 ± 3.4 | 0.3 ± 1.6 | 0.85 |
| Progesterone | 9.5 ± 2.2 | 7.4 ± 1.6 | 0.09 | 9.0 ± 2.5 | 9.0 ± 1.6 | 0.96 | 8.3 ± 1.7 | 9.5 ± 2.4 | 0.35 | 9.2 ± 1.8 | 8.8 ± 2.2 | 0.79 |
| Estrone | 158.7 ± 3.2 | 247.4 ± 2.7 | 0.10 | 189.2 ± 3.2 | 160.6 ± 3.1 | 0.55 | 184.7 ± 3.0 | 182.0 ± 3.3 | 0.95 | 191.0 ± 2.7 | 180.4 ± 3.3 | 0.82 |
| Estradiol | 41.1 ± 2.3 | 57.1 ± 1.9 | 0.07 | 53.0 ± 2.1 | 35.2 ± 2.3 | 0.04 | 47.3 ± 2.0 | 42.9 ± 2.5 | 0.54 | 48.5 ± 2.1 | 43.7 ± 2.3 | 0.54 |
| DHEAS | 80.6 ± 2.1 | 94.9 ± 1.9 | 0.32 | 80.5 ± 2.2 | 89.2 ± 2.0 | 0.56 | 83.6 ± 2.0 | 82.9 ± 2.2 | 0.95 | 79.5 ± 2.6 | 84.7 ± 1.9 | 0.74 |

Units: 27-hydroxycholesterol (nM), cholesterol (mg/dl), triglycerides (mg/dl), high-density lipoproteins (mg/dl), low-density lipoproteins (mg/dl), testosterone (ng/ml), progesterone (ng/ml), estrone (pg/ml), estradiol (pg/ml), DHEAS (µg/dl); Abbreviations: DHEAS, dehydroepiandrosterone sulfate; HT, hormone therapy

^a^ Welch's t-test for continuous variable [mean± std];

^b^ Based on menstrual-phase specific residuals; geometric mean corresponds to concentrations in the early follicular phase (mean value of samples collected in early follicular phase added to residual value as a constant)

Missing: testosterone=5, progesterone=5, estrone=8
